# Applications of Iron Oxide-Based Magnetic Nanoparticles in the Diagnosis and Treatment of Bacterial Infections

**DOI:** 10.3389/fbioe.2019.00141

**Published:** 2019-06-18

**Authors:** Chen Xu, Ozioma Udochukwu Akakuru, Jianjun Zheng, Aiguo Wu

**Affiliations:** ^1^Cixi Institute of Biomedical Engineering, Chinese Academy of Science (CAS) Key Laboratory of Magnetic Materials and Devices & Key Laboratory of Additive Manufacturing Materials of Zhejiang Province, Ningbo Institute of Materials Technology and Engineering, Chinese Academy of Sciences, Ningbo, China; ^2^Department of Experimental Medical Science, Hwa Mei Hospital, University of Chinese Academy of Sciences, Ningbo, China; ^3^Key Laboratory of Diagnosis and Treatment of Digestive System Tumors of Zhejiang Province, Ningbo, China; ^4^Department of Radiology, Hwa Mei Hospital, University of Chinese Academy of Sciences, Ningbo, China

**Keywords:** magnetic nanoparticles, bacterial infection, bacterial target molecules, detection, therapy

## Abstract

Diseases caused by bacterial infections, especially drug-resistant bacteria have seriously threatened human health throughout the world. It has been predicted that antimicrobial resistance alone will cause 10 million deaths per year and that early diagnosis and therapy will efficiently decrease the mortality rate caused by bacterial infections. Considering this severity, it is urgent to develop effective methods for the early detection, prevention and treatment of these infections. Until now, numerous efforts based on nanoparticles have been made to detect and kill pathogenic bacteria. Iron oxide-based magnetic nanoparticles (MNPs), as potential platforms for bacteria detection and therapy, have drawn great attention owing to their magnetic property. These MNPs have also been broadly used as bioimaging contrast agents and drug delivery and magnetic hyperthermia agents to diagnose and treat bacterial infections. This review therefore overviews the recent progress on MNPs for bacterial detection and therapy, including bacterial separation and enrichment *in vitro*, bacterial infection imaging *in vivo*, and their therapeutic activities on pathogenic bacteria. Furthermore, some bacterial-specific targeting agents, used to selectively target the pathogenic bacteria, are also introduced. In addition, the challenges and future perspective of MNPs for bacterial diagnosis and therapy are given at the end of this review. It is expected that this review will provide a better understanding toward the applications of MNPs in the detection and therapy of bacterial infections.

## Introduction

Diseases caused by bacterial infections have raised worldwide concern and the early diagnosis of such bacterial infections is of great significance for diseases therapy in the clinic (Váradi et al., 2017). The conventional diagnosis method to discriminate bacterial pathogens in the clinic often depends on the cultivation of bacteria, which is acknowledged as the “gold standard” in the clinical diagnosis of bacterial diseases (Pazos-Perez et al., 2016; Wohlwend et al., 2016; Xu et al., 2018). However, the traditional detection method consisting of cultivation, selective enrichment and conformation, is a tedious and time-consuming process; it takes up to 3 to 7 or more days to complete the biochemical testing process, delaying feedback to patients (Law et al., 2015; Váradi et al., 2017). To shorten the detection time and to acquire more accurate information about the bacteria, many more advanced methods including enzyme-linked immunoassays (Liu et al., 2017), western blotting (Liana et al., 2017), polymerase chain reaction (PCR) (Nguyen et al., 2017; Váradi et al., 2017), and whole genome sequencing (Ellington et al., 2017; Váradi et al., 2017), have been developed. The deficiencies of these modern methods are that they not only need precise and expensive instruments but also demand a lot from the operators (Sheikhzadeh et al., 2016; Wang et al., 2018a), consequently hindering their extensive use in the clinic.

For the past few years, iron oxide-based magnetic nanoparticles (MNPs) have extensively been studied as useful bacterial detection platforms due to their magnetic property (Shen et al., 2016; Suaifan et al., 2017; Yang et al., 2017; Wang et al., 2018b). Additionally, these MNPs have been widely used as bacterial separation agents (Shen et al., 2016; Xu et al., 2018), drug delivery (Bhattacharya and Neogi, 2017; Tokajuk et al., 2017; Wang et al., 2018c), bioimaging contrast agents (Lefevre et al., 2011; Li et al., 2017b) and magnetic hyperthermia agents (Ribeiro et al., 2018; Wang et al., 2018c) to diagnose and treat bacterial infections. For example, MNPs can be functionalized with target molecules such as various antibodies, antibiotics, antimicrobial peptides, bacteriophages and aptamers for bacterial separation and concentration (Chen et al., 2017). On the basis of the surface modification, MNPs conjugated with different metals allow the development of various methods for bacterial detection, including colorimetric, fluorescent, and surface-enhanced Raman detections (Yuan et al., 2018b). Apart from the *in vitro* detection methods, superparamagnetic iron oxide-based NPs have also been demonstrated as magnetic resonance imaging (MRI) contrast agents for *in vivo* bacterial imaging (Li et al., 2017b). Furthermore, MNPs with unique magnetic properties and high specific surface area have shown great promise in antibacterial applications (Lai and Chen, 2013; Ribeiro et al., 2018).

**Scheme 1:**
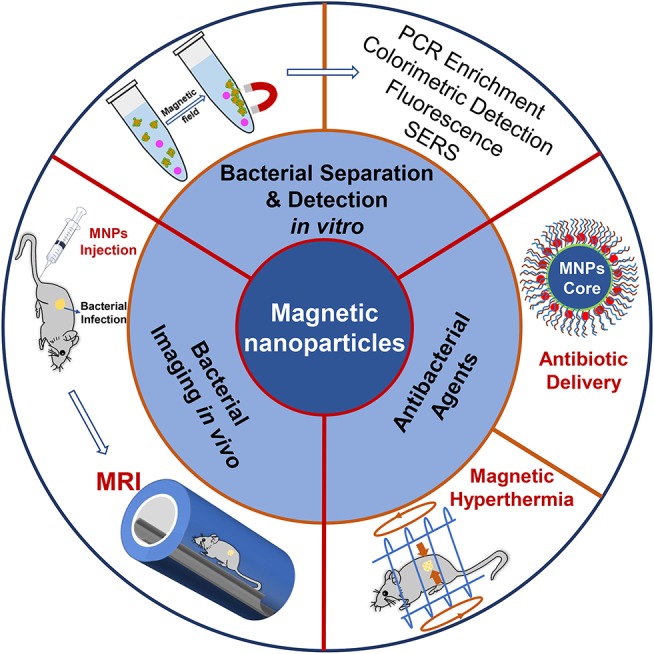
MNPs based nanoparticles for bacterial detection and therapy.

The diagnosis and treatment of bacterial diseases are of great concern for the prevention and control of bacterial infections. Figuring out the role of MNPs on bacterial diagnosis and treatment might have a guiding significance in designing and constructing MNPs-based materials for the detection and therapy of bacterial infections. This review therefore summarizes some recent progress on MNPs-based materials for bacterial detection and therapy, including bacterial detection *in vitro, in vivo* bacterial infection imaging, and their therapeutic activities on pathogenic bacteria (Scheme 1). First, target molecules for bacteria are listed, and their detection sensitivities as well as bacterial selectivity are summarized. Second, we present the available methods based on MNPs for *in vitro* and *in vivo* bacterial detection. Third, MNPs used as antibiotic delivery and magnetic hyperthermia agents for bacteria therapy are discussed. Lastly, the challenges and outlook of MNPs for bacterial diagnosis and treatment are put forward.

## Bacterial Target Molecules for Bacterial Separation and Enrichment

It was reported that centrifugation and filtration are commonly used for rapid bacterial separation and concentration (Liébana et al., 2013). Compared to the nonspecific methods, MNPs modified with bacteria-specific target molecules are quite suitable for bacterial separation and concentration because they can selectively target specific bacteria and can be controlled easily by an external magnetic field (Zhu et al., 2015). These emphasize their potential use in the rapid, efficient, specific capture, and enrichment of targeted bacteria from complex samples. The success of the separation and enrichment of designated bacteria by MNPs depends on the selection of target molecules. Previous studies have reported that various antibodies, antibiotics, antimicrobial peptides, bacteriophages as well as aptamers, which can be used as target molecules for bacteria, have been modified on the surface of MNPs for bacteria labeling and separation under a magnetic field (Chen et al., 2017). The representative target molecules for bacterial detection are listed in Table 1.

**Table 1:** Examples of bacterial target molecules conjugated MNPs for bacterial detection.

**Target agent**	**Materials**	**Detected bacteria**	**Sample**	**Method**	**Detection limit**	**References**
Antibodies	Anti-*E. coli* antibody conjugated MNPs	*E. coli*	Bacterial suspension	Fluorescence	10 CFU/mL	Park et al., 2017
	monoclonal antibody (MAb)-conjugated MNPs	*S. typhimurium*	Bacterial suspension	Colorimetric detection	2 × 10 cells	Shim et al., 2014b
	monoclonal antibodies (mAbs)-conjugated magnetic beads	*E. coli* and *F. tularensis*	Bacterial suspension	SERS and fluorescence	10^2^ cells/mL	Jang et al., 2016
Vancomycin	Fe_3_O_4_/Van/PEG magnetic nanocarrier; Van-PEG-PLL-MNPs	*L. monocytogenes*	Mixed solutions	PCR	10 CFU/mL	Zhu et al., 2015; Yang et al., 2017
	Van-PEGylated-MNPs	*L. monocytogenes*	Bacterial suspension	PCR	30 CFU/mL	Meng et al., 2017
	Fe_3_O_4_@Ag-Van MNPs and Au@Ag NPs	Broad range of Gram-positive and Gram-negative bacteria	Bacterial suspension	SERS	5 × 10^2^ cells/mL	Wang et al., 2018a
Vancomycin and ALP-IgG	ALP-IgG-Van- magnetic beads	*S. aureus*	Water/milk/urine and saliva	Fluorescence	3.3 CFU/mL	Yang et al., 2016
Streptavidin	MNP@Strep/Ag	*S. aureus* and *S. pyogenes*	knee joint fluid	SERS	–	Fargašová et al., 2017
Amoxicillin	Amoxicillin-conjugated Fe_3_O_4_	*S. aureus*/ *E. coli*	Mixed solutions	MALDI MS	10^3^ CFU/mL	Hasan et al., 2016
Antimicrobial peptide	AMP modified Fe_3_O_4_ NPs and 4-MPBA modified Au@Ag-GO nanocomposites	*E. coli, S. aureus* and *P. aeruginosa*	Whole blood	SERS	10 CFU/mL	Yuan et al., 2018a
T4 bacteriophage	T4 bacteriophage modified Fe_3_O_4_	*E. coli*	Bacterial suspension	–	–	Liana et al., 2017
T7 bacteriophage	T7 bacteriophage-conjugated magnetic beads	*E. coli*	Drinking water	Colorimetric detection	10 CFU/mL	Chen et al., 2015a
PAP1 bacteriophage	PAP1-functionalized magnetic beads	*P. aeruginosa*	Bacterial suspension	Colorimetric detection	2 × 10^2^ CFU/mL	He et al., 2017
E. coli specific DNAzyme	MNP-DNAzyme-AChE (MDA) complex and DNA-templated fluorescent silver nanoclusters	*E. coli*	Bacterial suspension	Fluorescence	60 CFU/mL	Zheng et al., 2018
Aptamers	Fe_3_O_4_-Ce6-Aptamer	*S. aureus*/ *E. coli*	Blood samples from mice	Fluorescence	10 CFU/mL	Wang et al., 2018b
	Aptamer modified Fe_3_O_4_ NPs and Co^2+^ enhanced N-(aminobutyl)-N-(ethylisoluminol) (ABEI) functional flowerlike gold nanoparticles	*S. typhimurium*	*In vitro*	Fluorescence	32 CFU/mL	Hao et al., 2017
	Aptamer-functionalized Fe_3_O_4_@silica	*S. aureus*	Whole blood	Fluorescence	682 CFU	Borsa et al., 2016

### Antibodies

Many studies have proven that antibodies specific to different bacteria can be conjugated on the surfaces of MNPs for the selective targeting and separation of bacteria. For instance, MNPs have been modified with H- or O-antibodies for the separation of *Salmonella typhimurium* (*S. typhimurium*), since the H- or O-antigens are recognized as the two typical surface structures of the *Salmonella* (Kuang et al., 2013; Sakudo et al., 2015; Kim et al., 2016). The H-antigen is the antigenic type of bacterial flagella while the O-antigen is a glycan polymer comprising lipopolysaccharides (LPS). The detection method for *S. typhimurium* was rapid and specific with neither the requirement of harmful reagents nor laborious pretreatments.

**Figure 1:**
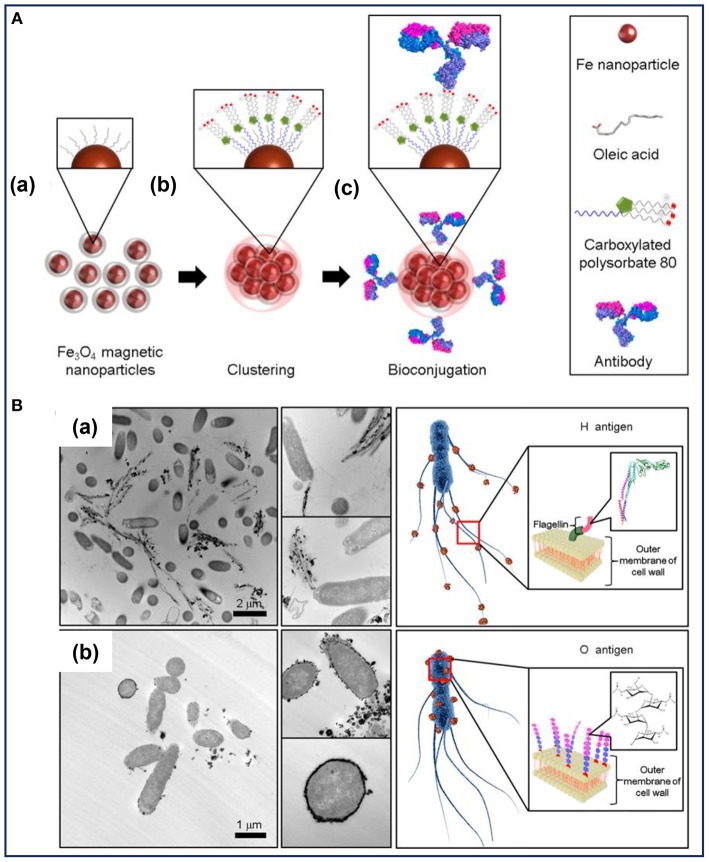
**(A)** Illustration of the preparation of the bioconjugated magnetic clusters with antibodies and **(B)** the different selective targets of *Salmonella typhimurium* for different antigens. Reprinted with permission from Kim et al. (2016). Copyright (2016) American Chemical Society.

To enhance the antibody immobilization at conjugation sites, MNPs clusters developed by the microemulsion method were used to highly select and rapidly separate *S. typhimurium* (Kim et al., 2016). As illustrated in Figure 1A, the MNPs coated with oleic acid were used as the precursor to form magnetic nanoclusters. Owing to the exposed carboxyl groups around the nanoclusters, they provided more conjugation sites for the immobilization of H- and O-antibodies. Consequently, the MNPs nanoclusters had the ability to effectively capture *S. typhimurium*. A difference in the cell separation efficiency was observed between the two antibodies-decorated nanoclusters, with a capture efficiency of about 99 and 57% for the O- and H-antibody-modified nanoclusters, respectively (Kim et al., 2016). Transmission electron microscopic analysis (Figure 1B) confirmed that the two different types of antibody-modified nanoclusters accumulated in different spatial locations of the bacteria according to the different antibody-antigen interactions. The H-antibody-modified nanoclusters accumulated in the bacterial flagella (Figure 1Ba), while the O-antibody-coated nanoclusters accumulated on the bacterial cell wall (Figure 1Bb). To explain the difference in the capture efficiencies, the authors put forward several potential reasons: on one hand, the mobility of the flagella as well as its easy detachment from the bacterial body hindered the binding interaction between the H-antibody and the corresponding antigen. On the other hand, the magnetic nanoclusters demonstrated a tendency to be adsorbed on biofilm containing polysaccharides and cellulose. As a type of glycan polymer, O-antigen on the cell wall membrane can capture more nanoclusters than that of the H-antigen. Accordingly, the O-antibody-coated nanoclusters showed high capture efficiency toward *S. typhimurium* (Kim et al., 2016). Therefore, the combined antibodies and MNPs-based nanoclusters led to a synergistic effect on the efficient and rapid detection of bacterial pathogens. The material design shows an inspiring strategy to improve bacterial capture efficiency by decorating MNPs with optimal types of antibodies. However, at very low concentrations of the detecting bacteria, the magnetic separation method still requires several hours to complete the enrichment steps.

A 3D microfluidic magnetic preconcentrator, in which an antibody was conjugated to the target molecule was fabricated to preconcentrate enterohemorrhagic *Escherichia coli* (*E. coli*) within 1 h (Park et al., 2017). By comparison, it can preconcentrate bacterial cells in large-volume samples. In general, antibody modified MNPs show potential as tools to capture, concentrate and discriminate different strains of bacteria. However, the high-cost and poor stability in severe environments remain the major drawbacks for the utility of antibodies.

### Antibiotics

Various antibiotics, including vancomycin, amoxicillin, streptavidin etc., have been extensively applied as agents for targeting bacteria. Vancomycin, which can bind to the bacterial cell wall of both Gram-negative and Gram-positive bacteria, was recognized as a target molecule for specific bacterial detection (Wang et al., 2014). As a glycopeptide antibiotic, vancomycin can recognize bacteria because of its ability to interact with the peptidoglycans on the bacterial cell wall. For Gram-positive bacteria, vancomycin can bind onto D-alanyl-D-alanine—the terminal residues of mucopeptides on the bacterial cell wall (Batchelor et al., 2010; Wang et al., 2014). For Gram-negative bacteria, vancomycin can bind with the L-lysin-D-alanine residues of peptidoglycans expressed on the outer membrane of bacterial cells (Kell et al., 2008; Wang et al., 2014). Thus, MNPs functionalized with vancomycin have been considered as an attractive type of capturing agent to effectively concentrate multiple strains of bacteria, such as *Staphylococcus aureus* (*S. aureus*) (Yang et al., 2016), *Bacillus subtilis* (*B. subtilis*) (Yang et al., 2016), and *Lister monocytogenes* (*L. monocytogenes*) (Wang et al., 2014, 2018a; Zhu et al., 2015; Yang et al., 2017). However, it is not efficient for the capture of vancomycin-resistant bacteria because of its poor affinity for the cell wall. Another antibiotic amoxicillin, which is a β-lactam antibiotic, can be attached to bacteria though penicillin binding proteins (PBPs). Thus, amoxicillin modified MNPs can also be used for the separation of bacteria (Hasan et al., 2016). The selective affinity of amoxicillin-functionalized MNPs toward bacteria mainly depends on the affinity of the β-lactam of amoxicillin with PBPs on the bacteria. Based on the binding mechanism, the amoxicillin modified MNPs were successfully applied to separate *S. aureus* and *E. coli*. The advantages of antibiotics are that they are highly stable, inexpensive and highly specific for most bacteria. Even so, it is generally thought that antibiotics are often small molecules and as such cannot provide a proper number of binding sites for bacterial identification. Ulteriorly, bacteria resistance issues have increased in recent years, necessitating the need to provide routes that avoids abusing antibiotics in bacteria detection.

### Antimicrobial Peptides

Antimicrobial peptides (AMP) enable the inactivation of different bacteria, viruses and fungi, making them remarkable as therapeutic agents for diseases. On account of the terrible increase of drug-resistant bacteria, the use of both synthetic and natural antimicrobial peptides has been explored for bacteria separation and detection (Da Costa et al., 2015). It has been demonstrated that antimicrobial peptides, such as bacitracin A- (Yuan et al., 2018a), pediocin- (Adhikari et al., 2014), cecropin- (Baek et al., 2016) functionalized MNPs can be applied as probes for bacteria capture, isolation and enrichment. Yuan et al. developed bacitracin A-modified MNPs and observed no change in the recognition site of the bacitracin A for bacteria, irrespective of its modification with MNPs. Owing to the direct interaction between bacitracin A and lipid, and indirect interactions mediated by Zn^2+^ and Na^+^, bacitracin A was tightly wrapped around the lipid pyrophosphate before and after the modification with MNPs, resulting in a strong interaction between bacitracin A and the bacteria. Thus, MNPs-bacitracin A conjugates would be attached to the bacteria, and the complexes would be easily separated under an external magnetic field (Yuan et al., 2018a). When applied as the bacteria capture element, antimicrobial peptides have several attractive advantages over antibodies: they are cost effective, possess better stability in harsh environments, and have long peptide chains, leading to a higher density of recognition sites for bacteria capture.

### Bacteriophages

Bacteriophages, which can specifically target bacteria without particular affinity for human host cells, have gained increased attention as an alternative to bacterial separation and detection. On the basis of the targeting and infectivity toward designated bacteria, bacteriophages have been used as recognition agents for bacterial detection. In this regard, T4 and T7 bacteriophages, which can infect *E. coli*, have been widely used to decorate MNPs for capturing *E. coli* (Chen et al., 2015a,b; Liana et al., 2017). Additionally, PAP1, a bacteriophage with high specificity for *Pseudomonas aeruginosa* (*P. aeruginosa*), was used to functionalize MNPs in order to establish a bacteriophage-affinity strategy for the separation and detection of *P. aeruginosa* (He et al., 2017). The PAP1-modified MNPs showed very high specificity toward *P. aeruginosa* without any response to the other interfering bacteria. The entire bacterial separation and detection process, including bacteria capture, PAP1 replication and bacteria lysis could be completed within 2 h (He et al., 2017). Interestingly, MNPs modified with bacteriophages can also be used to exclude the interference of inactive bacteria, since the bacteriophages only replicate in active bacterial cells (Chen et al., 2017; He et al., 2017). Furthermore, these kinds of MNPs modified with bacteriophages could be extensively used for the detection of other bacterial pathogens by utilizing virulent bacteriophages specific to target bacteria. Considering the relatively inexpensive and easy solution to obtain bacteriophages for different strains of bacteria, bacteriophages offer significant advantages for bacterial detection.

### Aptamers

As single stranded nucleic acids (DNA or RNA), aptamers have shown great potential in constructing recognition probes for bacterial detection. To sensitively detect the pathogenic bacteria, aptamers can be integrated with MNPs to construct a simple capture platform for bacteria (Shen et al., 2016; Wang et al., 2018b). When exposed to target bacteria, the corresponding aptamer will attach on the bacterial cells with high affinity and selectivity, and in contrast to antibodies, they can be exposed to elevated temperatures without being irreversibly denatured. Afterwards, the bacterial pathogens can be separated and further concentrated from their bloodstreams by an external magnetic field. It is worth noting that MNPs modified with different aptamers can be used to separate and concentrate different bacteria or other biomarkers. For instance, Wang and co-researchers reported a nanosystem based on aptamer functionalized MNPs for early diagnosis of blood disinfection (Wang et al., 2018b). Based on the nanoparticles, the multiple strains of bacteria could be successfully diagnosed, and bacterial strains identification and enrichment could be achieved in a single step. Aptamer-based capture platform (denoted as Apt-Fe_3_O_4_@mTiO_2_) has been constructed for the sensitive detection of *S. aureus* in bloodstream infections (Shen et al., 2016). The bacterial-capture efficiency of the Apt-Fe_3_O_4_@mTiO_2_ platform within 2 h was up to 80% even at low infectious doses. After the application of an external magnetic field, the *S. aureus* in the complexes could be selectively separated for further detection. It was demonstrated that MNPs conjugated with different aptamers could provide a feasibility for sensitive and specific detection of bacterial pathogens.

## MNPs-based Composites for Bacterial Detection *in vitro*

The ease of utilizing magnetic fields to control the location of MNPs-based composites after their conjugation with different bacterial target molecules has been explored for bacterial separation, enrichment, and discrimination. Thus, MNPs assisted with different detection methods, such as polymerase chain reaction (PCR), colorimetric detection, fluorescent detection, and surface-enhanced Raman detection (Yuan et al., 2018b) have been used to design various platforms for bacterial detection.

### PCR Enrichment

PCR is considered as one of the most promising alternatives to conventional methods of molecular diagnostics. However, it requires onerous steps and excessive labor for preconcentrating a relatively small number of bacterial cells from a liquid sample. To reduce the preconcentration time and extra steps, MNPs conjugated with different bacterial target molecules can enable bacterial cells to be concentrated prior to PCR in an integrated microfluidic PCR system, taking about 2 h or less to complete the preconcentration process and PCR steps (Ganesh et al., 2016). The system shows potential for an application as a platform for the rapid and specific detection of bacteria. To enhance the detection sensitivity of bacterial pathogens, Fe_3_O_4_@SiO_2_-based MNPs conjugated with *Pseudomonas aerugino*sa (*P. aerugino*sa) Genomic DNA (Tang et al., 2013), amino-rich silica-coated MNPs (Bai et al., 2016), MNPs conjugated with different antibodies for *S. aureus* and *Salmonella enteritidis* (*S. enteritidis*) (Houhoula et al., 2017), and MNPs functionalized with vancomycin (Meng et al., 2017) were used directly in the PCR enrichment procedure for bacterial pathogen enrichment. These studies demonstrated that the MNPs-based composites showed great potential for highly efficient enrichment of bacterial pathogens without time-consuming and onerous steps in the PCR procedure.

### Colorimetric Detection

**Figure 2:**
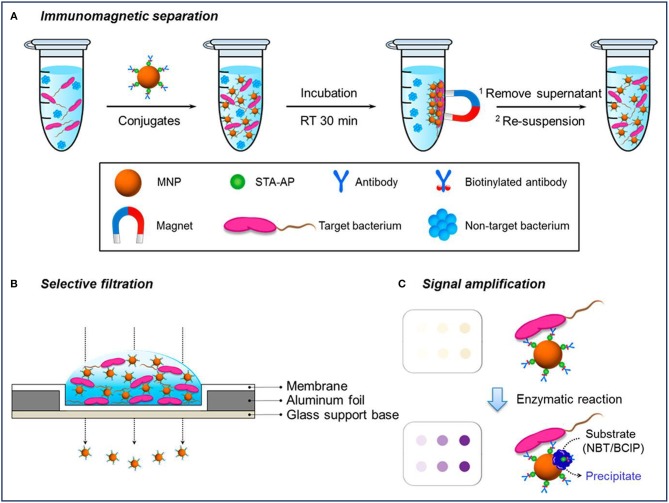
Schematic diagram of a selective filtration method for the colorimetric detection of *Escherichia coli*. Reprinted with permission from Kim et al. (2018). Copyright (2018) American Chemical Society. **(A)** Immunomagnetic separation for target bacteria using MNP conjugates; **(B)** selective filtration of target bacteria-conjugates complexes, and **(C)** colored spots on the filter membrane by target bacteria-conjugates (upper) and enhanced colorimetric spots by enzymatic amplification with precipitation on the bacteria-conjugate surfaces (lower).

Colorimetric detection is a qualitative analysis, which is based on bacteria-induced color changes which are visible to the naked eye. MNPs-based platforms have been developed for colorimetric detection of bacteria. Previous studies have reported that MNPs modified with monoclonal antibodies (mAb) were directly used to rapidly and sensitively detect *Salmonella typhimurium* (*S. typhimurium*) (Shim et al., 2014b) and *Listeria monocytogenes* (*L. monocytogenes*) (Shim et al., 2014a) based on color changes, which arose from MNPs aggregates through the filtration process. First, MNPs conjugated with the antibody were used to concentrate and purify the target bacterium under magnetic fields. After filtering through a membrane, the MNPs conjugates bound or unbound to bacteria can be easily separated by vacuum pressure, and the changes of color signals caused by the remaining MNPs reflected the amount of bacteria as shown in Figure 2 (Kim et al., 2018). For the *L. monocytogenes*, the capture efficiency of the conjugates ranged from 48 to 89% for solutions with bacterial cells from 2 × 10^3^ to 2 × 10^1^, and the results can be concluded within 35 min (Shim et al., 2014a). The efficiencies gradually decreased with an increase in the concentration of *L. monocytogenes*, which might be because of the limited addition of mAb-MNPs. It was observed that the bacterial capture efficiency would increase significantly, if more mAb-MNPs were used to test the bacterial solutions.

Gold (Au) NPs, which have distinct color changes caused by NPs aggregation, are the most common noble metal NPs to be used in colorimetric detection (Yuan et al., 2018b). As a consequence, MNPs-Au conjugates are promising platforms for bacterial detection. For example, Alhogail and others designed MNPs-coated Au conjugates for the detection of *L. monocytogenes*. After the surface modification of the gold sensor, the black MNPs covered up the color of the gold NPs. Upon cleavage of the peptide sequence by *Listeria* protease, the color of the conjugates changed from black to gold (Alhogail et al., 2016). Colorimetric method is very convenient and does not require any complicated equipment, and therefore could be used as a rapid, sensitive, and cost-effective tool for bacterial detection.

### Fluorescent Detection

**Figure 3:**
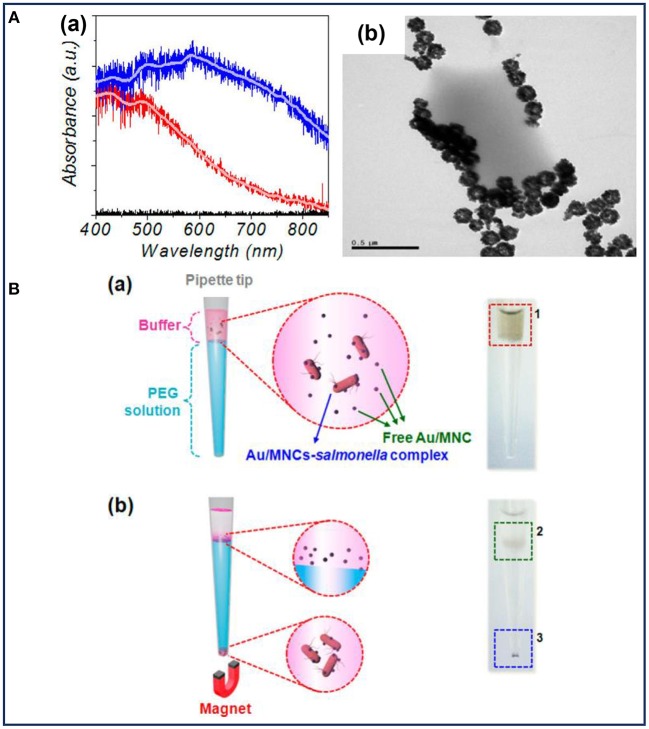
**(A)** Characterization of the Au-coated MNPs: (a) absorption spectra of MNPs (red line) and Au-coated MNPs (blue line), (b) TEM image of Au-coated MNPs bound with *Salmonella typhimurium*; **(B)** schematic illustration and the corresponding optical images before (a) and after (b) magnetophoretic chromatography. Reprinted with permission from Kwon et al. (2013). Copyright (2013) American Chemical Society.

Fluorescent detection is more sensitive and offers a higher detection limit than colorimetric methods. As a result of its low background, high sensitivity, high specificity, and the ease for quantitative analysis, fluorescent detection has been widely combined with MNPs for bacterial detection, since it is an emerging trend for the development of efficient biosensors for clinical use (Kwon et al., 2013; Tang et al., 2013; Chen et al., 2015c; Qin et al., 2016). MNPs with different fluorescent labels, such as Au (Kwon et al., 2013), rare earth-doped upconversion NPs (Qin et al., 2016), and the other labels (Jang et al., 2016; Shelby et al., 2017; Gontero et al., 2018) were conjugated with antibody or other target molecules for the detection of pathogenic bacteria. In these composite NPs, MNPs were used to capture and separate bacteria. Kwon et al. prepared Au-coated MNPs for the detection of *S. typhimurium* with the help of magnetophoretic separation (Kwon et al., 2013). The absorption of visible light could be observed after coating with Au NPs, indicating an enhanced sensitivity of the light absorption (Figure 3Aa). It was observed that after co-culturing with *S. typhimurium*, the Au-coated MNPs tended to accumulate around the bacteria (Figure 3Ab). As shown in Figure 3B, Au-coated MNPs-bacteria complex separated from the free Au-coated MNPs could move under an external magnetic field. The isolated Au-coated MNPs-bacteria complex at the bottom of each tube could be dispersed for further measurement by UV-vis spectrometer. This detection method based on magnetophoretic chromatography provided a detection limit of 100 CFU/mL for *S. typhimurium*.

Furthermore, the detection sensitivity can be improved by doping with other fluorescent labels (Kwon et al., 2013). Rare earth-doped upconversion NPs (UCNs) can also be used as the luminescence labels to detect bacterial pathogens since they have many attractive properties such as superior photostability, sharp emission lines and lack of autofluorescence. Consequently, MNPs have been combined with UCNs to construct a specific and sensitive platform to combine magnetic capture with fluorescent identification for the detection of *Porphyromonas gingivalis* (*P. gingivalis*) (Qin et al., 2016). The results showed that the platform, which comprised of magnetic and fluorescent modalities, allowed for the quantitative detection of pathogens in a wide range of concentrations.

### Surface-Enhanced Raman Detection

Surface-enhanced Raman spectroscopy (SERS) detection for bacteria can avoid the lengthy time for sample preparation and has been exploited for bacterial detection owing to the tremendous enhancement of Raman signal. In order to make the most of SERS for bacterial detection and monitoring, it is desirable to combine MNPs with SERS to construct a method for bacteria capture and detection (Liu et al., 2011). Recently, Fe_3_O_4_@Ag (Fargašová et al., 2017; Wang et al., 2018a) and Ag@Fe_3_O_4_ (Li et al., 2017a) composite MNPs have been constructed and modified with different target molecules such as vancomycin for a wide range of bacteria, biotinylated antibodies for both *S. aureus*, and *Streptococcus pyogenes* (*S. pyogenes*), for pathogenic bacteria separation and detection. Wang and co-researchers presented a sensitive MNPs-assisted SERS biosensor based on Fe_3_O_4_@Ag MNPs and Au@Ag NPs to effectively capture and discriminate bacteria. The Fe_3_O_4_@Ag MNPs were used as multifunctional platforms for bacteria capture and enrichment, as well as SERS substrates to enhance the signals of captured bacteria. An additional amount of the Au@Ag NPs was also introduced to further improve the SERS detection sensitivity. Since vancomycin has been reported to have a strong affinity for a broad range of Gram-positive and Gram-negative bacteria, this combined platform based on vancomycin modified Fe_3_O_4_@Ag MNPs and Au@Ag NPs could be applied for the detection of various strains of bacteria. It was demonstrated that both *E. coli* and methicillin-resistance *S. aureus* (MRSA) could be effectively captured by vancomycin-modified Fe_3_O_4_@Ag MNPs. Upon separating and rinsing the bacteria, the MNPs and Au@Ag NPs constructed a very large number of hot spots on the bacteria cells synergistically, leading to an ultrasensitive SERS detection with a low detection limit of 5 × 10^2^ cells/mL. More importantly, different bacteria such as *S. aureus, E. coli*, and MRSA could be sensitively and specifically discriminated according to different SERS spectra, and the results were further verified by the principal component analysis (PCA).

**Figure 4:**
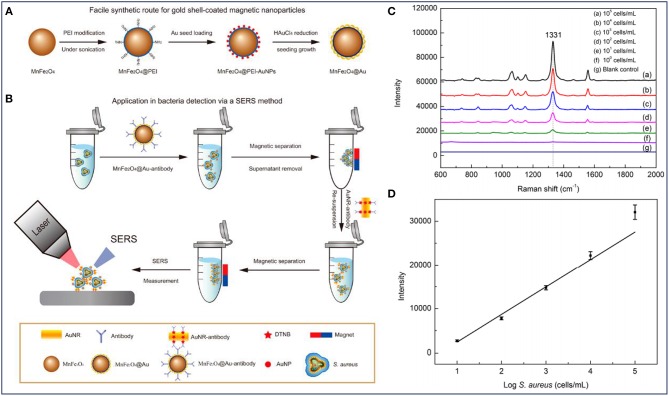
Au-coated magnetic NPs (AuMNPs) for *Staphylococcus aureus* capture, separation and detection. **(A)** Illustration of synthetic route of AuMNPs, **(B)** illustration of the detection procedures for *S. aureus* via a SERS method, **(C)** SERS spectra with various concentrations of *S. aureus* (from 10^1^ to10^5^ cells/mL), and **(D)** calibration curve for *S. aureus* at a concentration ranging from 10^1^ to 10^5^ cells/mL obtained by using SERS intensity at 1,077 cm^−1^. Reprinted with permission from Wang et al. (2016). Copyright (2016) American Chemical Society.

Previous studies have demonstrated that bacterial Raman spectra is directly related to bacterial cell wall components. Therefore, the differences in bacterial cell wall components make the SERS signals unique for different strains of bacteria. Based on these, the combined system shows great potential for the detection of bacterial infections. Furthermore, it was reported that the SERS intensity could also reflect the concentration of bacteria (Wang et al., 2016). As shown in Figure 4A, Au-coated core/shell magnetic NPs (AuMNPs) were designed, and further modified with *S. aureus* antibody and SERS tag for *S. aureus* capture, separation and detection (Figure 4B). The magnetic core endowed the nanocomposites with superior magnetic property for bacteria separation, and the outer Au shell provided high SERS activity for bacteria detection. According to the SERS spectra corresponding to different concentrations of *S. aureus*, several strong Raman bands of the SERS tag were observed (Figure 4C). It was obvious that with the help of SERS tag, *S. aureus* was detected with a detection limit of 10 cells/mL. Moreover, the main Raman peak (1331 cm^−1^) exhibited a linear relationship with the logarithm of bacteria concentrations ranging from 10^1^ to 10^5^ cells/mL (Figure 4D).

## Magnetic Nanoparticles as MRI Contrast Agents for Bacterial Detection *in vivo*

The common *in vivo* imaging methods for bacterial infections based on fluorophores, radioisotopes and microbubbles cannot provide enough information about the lesion site. Various studies have reported MRI as an alternative imaging modality for visualizing bacterial infection *in vivo* without ionizing radiation and invasion, since ^1^H is the most abundant magnetic nucleus in humans and is the most commonly investigated subject in MRI. This is complemented by the capacity of MRI to image the abnormal structures in a three-dimensional tomographic manner with a high spatial resolution. Previous studies reported that MNPs with superparamagnetism could shorten the longitudinal or transverse relaxation time of water protons nearby, thus MNPs-based contrast agents have been used to enhance the signal of the abnormal anatomy for the detection and monitoring of infectious diseases (Lefevre et al., 2011; Chen et al., 2016a). Particularly, MRI has been explored to noninvasively track bacteria and monitor antibiotic therapy of bacterial infection by using MNPs-based contrast agents (Lefevre et al., 2011; Hoerr et al., 2013).

To investigate the infection biology of clinically relevant bacteria, Hoerr and others established an imaging platform for *S. aureus* tracking *in vivo* by MRI. They constructed subcutaneous and systemic mouse infection models by the direct injection of MNPs-labeled or unlabeled *S. aureus*. After 24 h, MRI images showed a high resolution in both systemic and subcutaneous mouse infection model, which allowed for bacterial cells tracking and afforded information on the organ's morphology as well as the inflammatory response (Hoerr et al., 2013). By labeling different bacteria with MNPs, the established bacterial labeling strategy for MRI can also be applied for tracking the other infectious diseases to investigate infection biology.

Furthermore, MNPs for contrast-enhanced macrophage MRI *in vivo* have been used for the assessment of antibiotic therapy. In contrast to the conventional extracellular contrast agents such as gadoterate dimeglumine, the MNPs-based contrast agents undergo macrophage phagocytosis, which would bring a visible signal intensity change during the acute period and after antibiotic treatment (Lefevre et al., 2011). On the other hand, MNPs-based contrast agents provided an ultrasensitive imaging in *Mycobacterium tuberculosis* (*M. tuberculosis*) (Lee et al., 2012) and *Helicobacter pylori* (*H. pylori*) (Li et al., 2017b) infection. Li et al. illustrated this strategy for the specific capture of *H. pylori* in the gastric environment for the first time (Li et al., 2017b). By reversibly binding with peptidoglycan on the bacterial cell wall, the crab-like MNPs allowed for an accelerated aggregation of magnetic graphitic nanocapsules (MGNs), which allowed an easier capture of *H. pylori*. For the *in vivo* study, mice were treated with MGN and MGN@B-PEG, respectively, by intragastric administration. When the MNPs were intravenously injected into infectious mice model, the signal intensity in the granulomatous site showed an enhancement on the T_2_-weighted MRI images. Obviously, it was observed that *H. pylori* was difficult to be detected after MGNs injection because of the rapid elimination of the NPs. On the contrary, MGN@B-PEG was aggregated and retained in the mice abdomen for a prolonged period, thus enabling a stable T_2_-weighted imaging of gastric mucosa infected with *H. pylori* (Li et al., 2017b). Taken together, MNPs-based contrast agents might serve as promising diagnostic and bioimaging platforms for the *in vivo* detection and tracking of bacterial infections.

## Magnetic Nanoparticles as Antibacterial Agents

MNPs have been used in medical and pharmaceutical areas as drug delivery and hyperthermia agents for bacteria killing since the late 1970s (Sica de Toledo et al., 2018). Different nanostructures of MNPs have been reported as antibacterial agents to kill a range spectrum of bacteria species, including multidrug-resistant bacteria and bacterial biofilms with less damage to the human host cells (Sica de Toledo et al., 2018), and their minimum inhibitory concentrations (MIC) toward different bacteria or biofilms in previous studies have been summarized in Table 2.

**Table 2:** The minimum inhibitory concentrations (MIC) of various MNPs toward different bacteria of biofilms.

**Materials**	**Particle size (nm)**	**MIC (μg/mL)**	**Bacteria**	**Method**	**Antibiotic**	**References**
Fe_3_O_4_	4–10	10	*E. coli* biofilm	–	–	Thukkaram et al., 2014
			*S. aureus* biofilm			
			*P. aeruginosa* biofilm			
Fe_3_O_4_	≤ 18	100	*S. epidermidis*	–	–	Taylor and Webster, 2009
Ag/Fe_3_O_4_	20	3	*E. coli*	–	–	Ghaseminezhad and Shojaosadati, 2016
Fe_3_O_4_	10	9.2				
Fe_3_O_4_@PEG-Ag	20–25	16	*E. coli*	–	–	Zomorodian et al., 2018
			*S. aureus*			
Fe_3_O_4_@PAA	10 ± 2	8000	*P. fluorescens*	Magnetic hyperthermia	–	Rodrigues et al., 2013
Fe_3_O_4_@APTES	17	100	*B. subtilis* biofilm	–	–	Ranmadugala et al., 2017
CoFe_2_O_4_	16 ± 5	50	*E. coli*	–	–	Venkatesan et al., 2015
Fe_3_O_4_-TiO_2_	–	12.5	*E. coli*	Simulated solar irradiation	–	Ma et al., 2015
			*S. aureus*			
MNP-CSA-13	14 ± 2	1	*P. aeruginosa*	–	–	Niemirowicz et al., 2015
VancomycinPEG-chitosan-MnFe_2_O_4_	25	0.61	*S. epidermitis*	–	Vancomycin	Esmaeili and Ghobadianpour, 2016
		0.78	*S. aureus*			
		0.78	*B. subtilis*			
		0.98	*MRSA*			
		39.06	*E. coli*			
		78.12	*P. aeroginosa*			
MNPs@Ag@HA	~40	200	*S. aureus* biofilm	Magnetic field	Gentamicin	Wang et al., 2018d
MnFe_2_O_4_@PrBrT	10	8	*E. coli*	Magnetic hyperthermia	–	Pu et al., 2016
		8	*S. aureus*			

Upon conjugating with different antibiotics such as vancomycin (Lai and Chen, 2013), gentamicin (Bhattacharya and Neogi, 2017), methicillin (Geilich et al., 2017) and cephalexin (Rayegan et al., 2018), MNPs and their derivatives (Au coated, Ag coated, Co doped, or cationic polymer modified) have been widely investigated for their potential to penetrate into bacteria cells and biofilm mass, which may inactivate bacteria and antibiotic-resistant bacteria (Niemirowicz et al., 2014, 2015; Venkatesan et al., 2015; Chen et al., 2016b; Pu et al., 2016; Zomorodian et al., 2018). Geilich et al. established highly organized methicillin-resistant biofilms on glass coverslips, and subsequently treated them with MNPs with and without an external magnetic field. After incubation for 24 h, the MNPs could penetrate into the robust biofilms in the presence of a magnet, while minimal iron penetration was observed in the absence of any magnetic field (Geilich et al., 2017). Furthermore, they demonstrated the penetration depth and antibacterial property of the MNPs loaded with methicillin. By using laser scanning confocal microscopy of the bacterial biofilms stained with Live/Dead Biofilm Viability kit, it was verified that the antibiotic delivery system constructed with MNPs could deepen the drug penetration as well as deliver high concentrations of antibiotics into the multiple layers of the biofilms, while the antibiotic alone could only control the planktonic bacteria without the ability to penetrate biofilms (Geilich et al., 2017). Consequently, the MNPs delivery system showed great potential as magnetic drug delivery system, which can control the movement and location of antibiotics, resulting in a rapid, and efficient treatment of biofilms.

**Figure 5:**
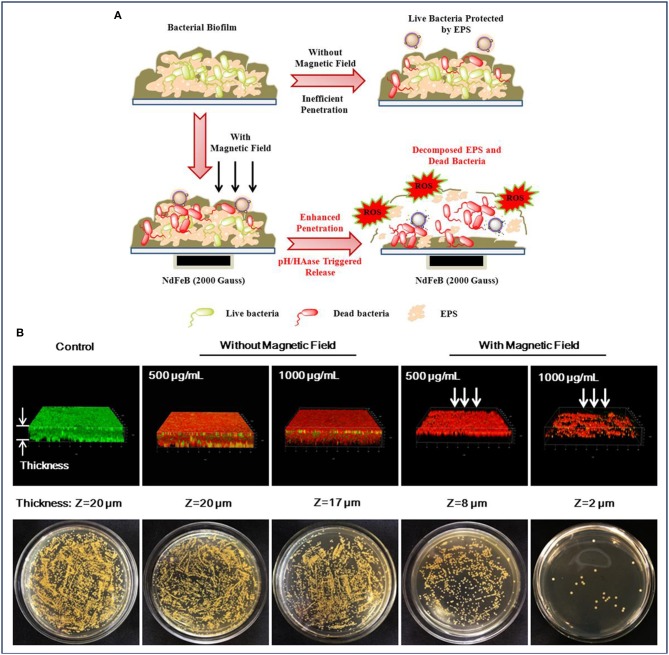
MNPs for enhanced biofilm distribution under magnetic field. **(A)** Illustration of MNPs based antibiotic and Ag delivery for inactivating the embedded bacteria with or without an external magnetic field, and **(B)** Live/dead staining of 3D reconstructions of *S. aureus* biofilm and bacterial colonies of surviving *S. aureus* in biofilms after treatment without and with an applied magnetic field, respectively. Reprinted with permission from Wang et al. (2018d). Copyright (2018) American Chemical Society.

Wang et al. also conducted the antibiofilm activity assay by treating the biofilms with MNPs, and similar results have been obtained. To further understand the behaviors of biofilms treated with the nanocarrier, the authors presented a probable mechanism, collected and analyzed the CLSM 3D images of the biofilms. As illustrated in Figure 5, a powerful nanocarrier based on MNPs for antibiotics and Ag NPs delivery could be guided to penetrate into *S. aureus* biofilm and significantly enhance the biofilm disruption (Wang et al., 2018d). When there was no external magnetic field, the intact and dense biofilm hindered the nanocarrier from penetrating into the biofilm, resulting in an insufficient antibiofilm efficiency (Figure 5A). It can be observed in Figure 5B that the number of dead bacteria increased significantly after treatment with the nanocarrier. However, the structure of the biofilm seemed to be compact and high amounts of live bacteria were protected by the extracellular polymeric substances (EPS) of bacteria. In contrast, the presence of an external magnetic field facilitated a deeper penetration of the nanocarrier into the established biofilms of *S. aureus*. It was illustrated in Figure 5A that under the acid environment caused by *S. aureus*, the nanocarrier was degraded and allowed the release of the antibiotics and Ag ions to the surrounding. Subsequently, the Ag ions also induced the production of intracellular reactive oxygen species (ROS), which accelerated the decomposition of EPS and further promoted the penetration of antibiotics into the biofilms. Notably, it can be seen in Figure 5B, after treatment with the nanocarrier under an external magnetic field, the number of live bacteria decreased dramatically and the thickness of biofilm also decreased compared with that of untreated group as well as the treated group without magnetic fields (Wang et al., 2018d).

Recently, MNPs were used as hyperthermia agents to treat bacterial infections which show more temperature susceptibility than human healthy host cells (Sica de Toledo et al., 2018). When placed under an alternating magnetic field with high frequency and amplitude, MNPs would absorb electromagnetic radiation and subsequently convert the magnetic energy to localized heat. Conjugated with different bacterial target molecules, these MNPs can specifically target the bacteria site and homogenous heat under alternating magnetic fields. The magnetic hyperthermia process will result in an enhanced membrane permeability and antibacterial property, since most bacterial pathogens will become vulnerable at an environmental temperature around 45°C or higher (Ibelli et al., 2018). It was also confirmed by Rodrigues and others that the bacterial morphology, viability and mechanical properties of *Pseudomonas fluorescens* (*P. fluorescens*) could be affected significantly by temperatures above 45°C. In order to study the effect of the magnetic heating on cell viability, planktonic *P. fluorescens* cells and biofilms were cultured on silicone coupons, and then were transferred to the hyperthermia equipment. After exposure to an external alternating magnetic field, the viability of both planktonic and biofilm cells decreased with the increasing temperature. According to CLSM and SEM, an increasing amount of dead bacterial cells was observed as the temperature increased, and most of the dead cells in the biofilm tended to be planktonic. Additionally, compared with the same direct heating temperatures, magnetic hyperthermia caused by MNPs resulted to a greater destruction of the bacterial biofilms (Rodrigues et al., 2013). Kim et al. further verified the antimicrobial efficacy of the magnetic hyperthermia in a mouse infection model caused by *S. aureus* (Kim et al., 2013). The MNPs-based hyperthermia agent was prepared by conjugating MNPs with biotinylated anti-protein A mAb for targeting *S. aureus in vivo*. After the injection of the MNPs conjugates, the infected mouse was placed under an alternating magnetic field with high frequency and amplitude. Subsequently, the remaining *S. aureus* was monitored by a luminescence method. The results showed that antibody modified MNPs had an enhanced antibacterial efficiency of about 80% against *S. aureus* under the alternating magnetic field. Therefore, MNPs based hyperthermia agents can be used as a kind of efficient antibacterial agents for the treatment of bacteria and biofilms.

Furthermore, the antimicrobial activity caused by magnetic hyperthermia can be improved by modifying MNPs with cationic polymers (Pu et al., 2016) or antibiotics (Nguyen et al., 2015; Wang et al., 2018c; Zomorodian et al., 2018). The probable direct reason is that the modification leads to a stronger interaction between the MNPs and the bacterial surface. When grafted with “soft” polycarbonate as the shell, the “hard” superparamagnetic core was afforded with greater charge density, leading to a stronger interaction between the MNPs and bacteria. As such, more bacterial cell membranes could be disrupted with the effect of magnetic hyperthermia under an external magnetic field. Thus, the structural integration of MNPs with cationic polymers brought about a synergistic destructive effect on bacterial cells (Pu et al., 2016). Fang and co-researchers combined magnetic hyperthermia with vancomycin to treat peri-implant osteomyelitis in rats' infection model (Fang et al., 2017). After the establishment of osteomyelitis model in rats, vancomycin and MNPs were injected intramuscularly, and the therapeutic effect was evaluated by incubating the specimens from the subcutaneous tissue and the implant site. Under an external magnetic field, MNPs conjugates could be heated up to 75°C. This high temperature enhanced the bacterial killing efficiency of vancomycin against methicillin-sensitive *Staphylococcus aureus* (MSSA). Meanwhile, the magnetic hyperthermia could also destroy the protection effect of biofilm on bacteria, leading to a deeper antibiotic penetration into the mature biofilm and an effective antibiotic delivery for the eradication of MSSA (Fang et al., 2017). Consequently, MNPs can be applied as drug delivery systems as well as magnetic hyperthermia agents for the synergistic therapy of bacterial infection.

## Conclusion and Outlook

In this review, MNPs were showcased as potential platforms to detect and treat bacterial infections. Upon the conjugation of different bacterial target molecules with MNPs, the conjugates demonstrate the ability to selectively attach on the surface of bacterial pathogens, showing their great potential as bioimaging contrast agents, drug delivery and hyperthermia agents for bacterial detection and therapy. However, several challenges still need to be overcome. For *in vitro* bacterial detection, most bacterial target molecules such as antibodies and bacteriophages are specific to one or some types of designated bacterium strains. If there are several different and uncertain bacteria strains to be tested, it is difficult to choose accurate target molecules to discriminate the different bacteria strains simultaneously. Thus, the detection selectivity needs to be further improved through different modification strategies. Additionally, most of the *in vitro* bacterial detection experiments were carried out and verified in simplified bacterial fluids, and further experiments should be conducted in complex biological fluids to demonstrate their sensitivity, specificity, and validity. For bacterial imaging *in vivo*, how to endow the NPs with the ability to discriminate infections caused by different bacteria strains remains a big challenge. Finally, there are few studies on MNPs with both detection and therapeutic features for bacterial infections, necessitating more research into the construction of multifunctional MNPs with the utility of imaging-guided treatment of bacterial infection since they have both imaging and therapeutic properties.

## Author Contributions

JZ and AW conceived the review paper proposal. CX wrote the manuscript draft. OA improved the quality of language. All authors revised the manuscript.

### Conflict of Interest Statement

The authors declare that the research was conducted in the absence of any commercial or financial relationships that could be construed as a potential conflict of interest.
